# Vincent's Angina

**Published:** 1919-09-20

**Authors:** 


### VINCENT'S ANGINA.

### A Note of Recent Observations.

During the past year considerable attention has been given to this condition, which, as Watson-Williams suggests, is preferably termed Vincent's disease, in that it is quite erroneous to consider it as limited to the throat alone. On the whole, one many conclude that the infection is much more prevalent than was at one time supposed, and also that it must be treated as a serious illness, having, on occasion, a fatal result.

In the first place, there is evidence to dispose of the formerly prevailing belief that in Vincent's angina there is more often than not a positive Wassermann reaction. At the Queen Alexandra's Military Hospital Taylor and McKinstry went very thoroughly into the bacteriology and serology of the condition, basing their observations on the examination of some three hundred cases of fuso-spirillary infection, of which about fifty were typical Vincent cases. Their chief conclusions were that the Wassermann reaction yielded no observable complement fixation test, except in one or two instances where there was a previous history of syphilis, and that a differentiation from the venereal condition could be readily made. Also, they state, that if in a Vincent's case the W.R. be positive, a double infection undoubtedly exists, and from the treatment point of view this, if true, should be very carefully remembered.

### Generalised Infection.

In evidence of generalised infection we read of several carrier cases, which are described as showing, at intervals, apart from increased local ulceration, generalised skin lesions and constitutional trouble. Usually, however, the chronic type of ulcers may persist for a long time without causing such outbursts, and in these it is very common for their true cause and bacteriology to be entirely missed. To illustrate the fact of their potential danger we may quote the case, described by Greely, of a doctor suffering from a liability to chronic septal ulcer. The patient developed, two months before coming under observation, a heavy membranous ulceration of his left tonsil and a generalised purpuric rash. A bacteriological diagnosis being made, a vaccine prepared from the fusiform bacilli was applied. At the first dose a marked local reaction occurred; at the second a similar reaction, but on this occasion accompanied by increased purpuric rash also. The vaccine was then suspended, but later a severe bronchitis supervened, with acute general symptoms, and large numbers of fusiform bacilli and spirilla in the sputum. A few days later the patient died, leaving good evidence for considering the result to be due to Vincent's angina infection.

A number of such multiple cutaneous cases have been recorded, and, although in the actual skin lesions, *i.e.*, bullæ, pustules, etc., only the common cocci of suppuration have been found, it is more than probable that the rarer infection is the underlying cause, particularly in view of the very definite reaction which vaccines are known to give in these instances.

### Persistence of Infection.

Our own experience has been that Vincent's organisms are particularly liable to remain in the crypts and remoter passages, even after vigorous local measures have apparently cleared up the case. In one instance, after an apparent local cure, the tonsils were enucleated and subjected to bacteriological examination. The deeper crypts were seen definitely to harbour the germs, giving thus a clear illustration of the principle that we should consider a previously-infected throat most liable to harbour the Vincent's organism. It should be impressed upon the practitioner that complications, and severe ones, may arise, and that the organism is by no means easily and finally cleared from the system.

### Two Main Conditions.

There are two main conditions to consider, namely, the appearance of (*a*) constitutional symptoms, and (*b*) excessive ulceration. In either case it is probably best for future developments to resort immediately to moderate doses of neo-salvarsan. There is not general agreement on this point, but to us such a high percentage of cases appear to benefit immediately from this intravenous attack on the infection that it seems the obvious method, unless the general condition of the patient absolutely contraindicates it. Hubbard considers that in ulcerative cases the extent of the necrosis often accounts for the futility of local applications, the objection to strong antiseptics being that they may gradually extend the area of invasion by destroying normal marginal tissue. Most of his fatal cases apparently showed this deep ulceration, and in view of the possible hopelessness of local measures in such instances, the intravenous method seems to be far preferable. Excision of necrotic areas, scraping, curetting, and their variations are all drastic measures which may be used with discretion, but are, in our opinion, not a little dangerous in causing further extension in unfavourable cases. It is probably best, as suggested by Carroll, that all tonsillar crypts and mucous pockets should be opened up to promote drainage, but without removal of tissue. This writer suggests applications of mono-chlor-acetic acid, iodine, chloride of iron in glycerin or nitrate of silver to all such localities after they have been thoroughly dried out with cotton-wool.

The latest views tend on the whole to make us fear Vincent's disease more seriously than we have done in the past, and in its study there are, as with most mouth conditions, points of the greatest interest.

